# VV-ECMO–supported management of severe ARDS secondary to melioidosis sepsis: A case report and concise review

**DOI:** 10.1016/j.idcr.2026.e02612

**Published:** 2026-05-15

**Authors:** Fengyun Wang, Chengzhi Xie, Mingrui Zhao, Yuxiang Pan, Yuxiang Xie, Xiaozhi Wang, Weiwei Zhu, Yiqiang Xie

**Affiliations:** a Department of Critical Care Medicine, Second Affiliated Hospital of Hainan Medical University, Haikou, China; b NHC Key Laboratory of Tropical Disease Control, Hainan Medical University, Haikou, China; c Department of Intensive Care Unit, Binzhou Medical University Hospital, Binzhou 256600, China; d Traditional Chinese Medicine department, Hainan Health Vocational College, Haikou, China

**Keywords:** Melioidosis, Burkholderia pseudomallei infection, Sepsis, Acute respiratory distress syndrome ARDS, Venovenous extracorporeal membrane oxygenation VV-ECMO, Metagenomic Next-generation sequencing mNGS

## Abstract

Melioidosis, caused by *Burkholderia pseudomallei (B. pseudomallei)*, is a life-threatening tropical infection that is frequently underdiagnosed because of its heterogeneous and nonspecific clinical presentation. We report a critically ill patient from an endemic area who developed fulminant pneumonia that progressed to septic shock and severe acute respiratory distress syndrome. Despite empirical broad-spectrum antimicrobial therapy, respiratory failure worsened, prompting early etiologic investigation with metagenomic next-generation sequencing, which identified *B. pseudomallei* and was subsequently confirmed by culture. The patient required early venovenous extracorporeal membrane oxygenation (ECMO) for refractory hypoxemia. Management included a targeted antimicrobial therapy in accordance with current guidelines and CT-guided drainage of a pulmonary abscess as definitive source control. The patient achieved full recovery without recurrence at follow-up. Early identification of the causative pathogen and timely source control were central to the management of melioidosis-associated severe ARDS. Advanced supportive measures, including ECMO, may be considered in selected patients with refractory hypoxemia as part of management involving multiple specialties.

## Highlights

• Melioidosis-associated ARDS may resemble severe community-acquired pneumonia.
• mNGS results require interpretation within clinical and epidemiological context.
• VV-ECMO may be considered for refractory hypoxemia in selected cases.
• Source control and prolonged antimicrobial therapy reduce the risk of relapse.

## Introduction

Melioidosis is a severe infectious disease caused by the Gram-negative bacterium *Burkholderia pseudomallei* (*B. pseudomallei*). It is an environmental saprophyte commonly found in soil and surface water in tropical and subtropical regions, and human infection occurs mainly through percutaneous inoculation, inhalation, or ingestion [1]. The disease is endemic in Southeast Asia and northern Australia [1], and in China, Hainan Province is recognized as an endemic region. Model-based estimates suggest that approximately 165,000 cases of melioidosis occur globally each year, resulting in around 89,000 deaths [2]. This level of disease burden is comparable to that of several infections recognized by the World Health Organization (WHO) as neglected tropical diseases [2].

More than 80% of patients with melioidosis have at least one underlying risk factor, with diabetes mellitus being the most common, and associated with an approximately 13-fold increased risk of infection [3]. Hazardous alcohol consumption, chronic kidney disease, and occupational exposure to soil or water are also recognized as important risk factors [1]. Extreme weather events, including typhoon-associated heavy rainfall and flooding, may increase environmental exposure and contribute to case clustering.

Clinically, melioidosis is known as “the great mimicker” because of its highly variable presentations and frequent misdiagnosis as tuberculosis or other community-acquired infections [4]. Severe disease commonly presents as community-acquired sepsis or pneumonia and is associated with mortality rates exceeding 40% in resource-limited settings [2]. The lungs are the most commonly affected organ (approximately 50% of cases), and in some patients, pulmonary infection can progress to acute respiratory distress syndrome (ARDS) and multiple organ failure, which are major contributors to mortality.

In cases of refractory hypoxemia that do not respond to conventional mechanical ventilation, extracorporeal membrane oxygenation (ECMO) may be considered as a rescue therapy [5]. We describe a patient with severe acute respiratory distress syndrome (ARDS) secondary to B. pseudomallei infection who received early venovenous ECMO support and subsequently recovered with management involving multiple specialties. This report provides additional clinical information relevant to the diagnosis and management of severe melioidosis.

## Case presentation

### Patient information

The patient was a 39-year-old male truck driver who had been unemployed for the previous six months and resided in Changjiang County, Hainan Province. He had a history of chronic alcohol abuse for more than ten years, but no other known chronic illnesses.

### Clinical findings

The patient initially presented to a local hospital with progressively worsening cough, fever, and dyspnea. He was diagnosed with severe pneumonia, septic shock, and multiple organ dysfunction syndrome (MODS: heart, lung, and kidney involvement). His Acute Physiology and Chronic Health Evaluation II (APACHE II) score at admission was 27, correlating with a predicted mortality rate of approximately 60%. Despite receiving 2.5 days of intensive treatment—including broad-spectrum antibiotics (meropenem 2 g every 8 h and levofloxacin 0.5 g once daily), invasive mechanical ventilation, and prone positioning—the patient’s condition continued to worsen. He developed refractory hypoxemia that did not respond to conventional therapy, with a sustained PaO₂/FiO₂ ratio below 100 mmHg even under 100% FiO₂, fulfilling the Berlin definition criteria for very severe ARDS.

In view of the patient’s clinical deterioration, our ECMO team initiated VV-ECMO cannulation at the referring hospital on June 23, 2025, and subsequently transferred the patient to our intensive care unit (ICU). Upon ICU admission, the patient’s APACHE II score had increased to 37, corresponding to a predicted mortality risk exceeding 85%, consistent with further clinical deterioration. The patient was in shock, with vital signs reflecting severe physiological compromise: temperature 39.2°C, pulse 170 beats/min, and blood pressure 115/62 mmHg (maintained with norepinephrine 0.8 μg/kg/min plus metaraminol 6 mL/h). Laboratory findings were consistent with progression of multiple organ dysfunction, including lactic acidosis (lactate 4.7 mmol/L), acute kidney injury (creatinine 324 μmol/L), and coagulopathy consistent with disseminated intravascular coagulation (DIC). On examination, the patient was sedated and intubated, with diffuse bilateral crackles on auscultation.

### Treatment timeline

**Fig. 1:**
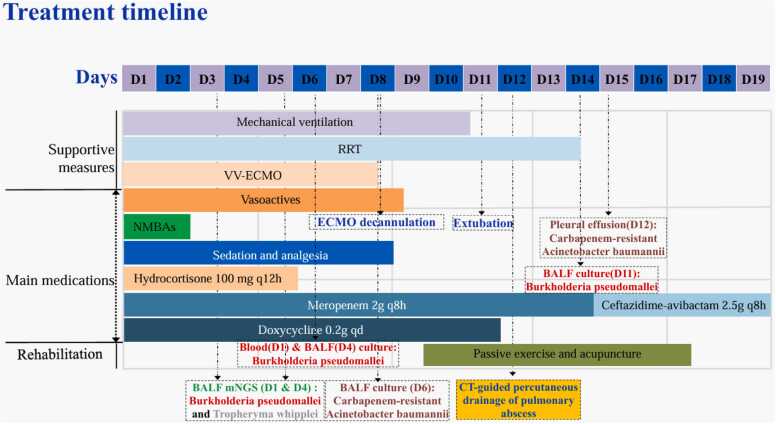
Clinical course and key interventions during the first 19 days of hospitalization. This timeline details the patient's clinical course, chronologically mapping the use of critical interventions. It documents the duration of major organ support—including mechanical ventilation, renal replacement therapy (RRT), and venovenous extracorporeal membrane oxygenation (VV-ECMO)—alongside the administration of vasoactive drugs, neuromuscular blockers, and sedative-analgesic agents. The evolution of the antimicrobial regimen is shown, beginning with meropenem and doxycycline and later transitioning to ceftazidime/avibactam. Key diagnostic milestones, such as the timing of metagenomic next-generation sequencing (mNGS), the return of culture results, and the percutaneous drainage of the pulmonary abscess, are indicated to provide context for therapeutic decisions. Abbreviations: ARDS, Acute Respiratory Distress Syndrome; BALF, Bronchoalveolar Lavage Fluid; CT, Computed Tomography; D, Day; mNGS, metagenomic Next-Generation Sequencing; NMBAs, Neuromuscular Blocking Agents; RRT, Renal Replacement Therapy; VV-ECMO, Veno-Venous Extracorporeal Membrane Oxygenation.

The key clinical events from admission to our ICU, diagnosis, and treatment to recovery and discharge are summarized in Fig. 1.

### Diagnostic assessment

Upon admission, the patient was initially diagnosed with severe pneumonia**,** very severe acute respiratory distress syndrome (ARDS)**,** sepsis**,** septic shock**,** MODS (heart, lung, kidney, liver), and DIC. Key clinical and laboratory parameters during the early hospital course are provided in the Supplementary File.

Establishing the microbiological diagnosis was a key challenge. On Day(D) 3 after admission (D1 for specimen submission), metagenomic next-generation sequencing (mNGS) of the patient’s bronchoalveolar lavage fluid (BALF) identified two potential pathogens: *Tropheryma whipplei* (*T. whipplei*, sequence reads: 29,975 copies) and *B. pseudomallei* (sequence reads: 22 copies). Although T. whipplei had a higher read count, it is typically associated with chronic infection [6], which was not consistent with the patient’s acute and rapidly progressive presentation. In contrast, B. pseudomallei is a recognized cause of acute severe pneumonia and sepsis [2], including reported cases in tropical regions such as Hainan.

**Fig. 2:**
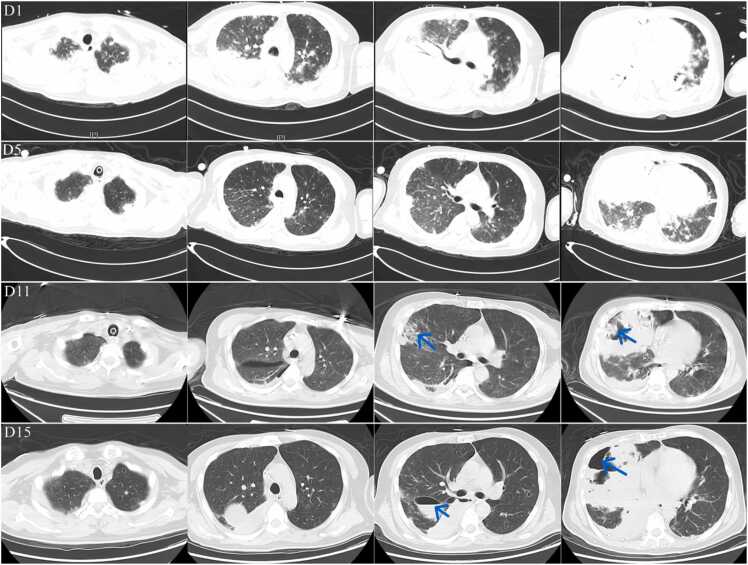
Progression of Pulmonary Findings on Serial Computed Tomography (CT). These representative axial CT images, obtained on days 1, 5, 11, and 15 after admission, are displayed in chronological rows. Each column corresponds to a consistent anatomical level, allowing for direct comparison of pathological changes in the upper, middle, and lower lung zones over time. D1 (top row): Extensive, diffusely distributed ground-glass opacities and consolidations are observed in both lungs, partially coalescing into a “white lung” appearance, most prominent in the posterior and basal regions. These findings are consistent with the radiological features of very severe ARDS. D5 (second row): After initiation of VV-ECMO and antimicrobial therapy, there was partial resolution of the diffuse infiltrates with some improvement in aeration, though extensive opacities and consolidations persisted. D11 (third row): While most infiltrates continued to clear, the improvement was asymmetric. A new cavitary lesion with thick walls, an air–fluid level, and surrounding consolidation emerged in the right middle and lower lobes (arrow), a finding highly suggestive of a developing lung abscess. D15 (bottom row): After percutaneous drainage of the abscess under CT guidance, the cavity was substantially smaller, with thinner walls and less surrounding inflammatory infiltration. The continued resolution of other pulmonary opacities signified marked radiological improvement.

The patient lived in a coastal area and had recent exposure to typhoon-associated conditions, both of which are recognized risk factors for melioidosis [7]. Based on the clinical presentation and epidemiological context, B. pseudomallei was considered the most likely causative pathogen. The diagnosis was subsequently confirmed by blood culture on D1 and BALF culture on D4 (reported on D6), both of which yielded *B. pseudomallei*. Chest computed tomography (CT) demonstrated the progression of pulmonary involvement (Fig. 2). At admission, CT showed diffuse bilateral infiltrates with consolidation, consistent with severe ARDS, including diffuse opacification of both lungs. Follow-up CT on D11 showed the development of an abscess in the middle and lower lobes of the right lung.

### Therapeutic intervention

The patient received comprehensive intensive care involving multiple therapeutic strategies tailored to his clinical condition.

Due to refractory hypoxemia, VV-ECMO was initiated by our ECMO team at the referring hospital via bedside cannulation. After transfer to our institution, VV-ECMO support was continued for a total of eight days. This intervention was critical not only for restoring systemic oxygenation but also for enabling a strategy of ultra-lung-protective ventilation during the first six days to facilitate pulmonary recovery. This “lung rest” approach employed extremely low tidal volumes (3 mL/kg predicted body weight) and low plateau pressures, aiming to minimize driving pressure and thereby reduce the risk of ventilator-induced lung injury (VILI). Our strategy was informed by robust evidence demonstrating that minimizing driving pressure during ECMO is associated with reduced mortality, making it a key modifiable factor for improving patient outcomes [8].

**Fig. 3:**
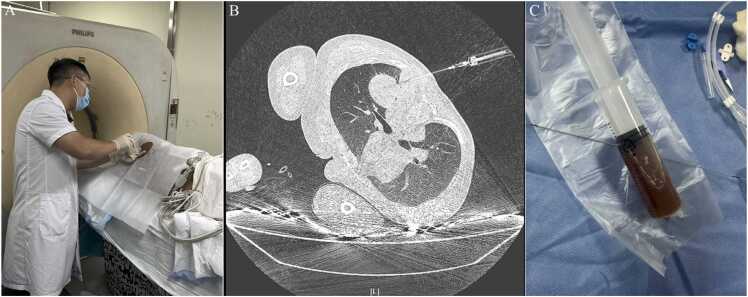
Percutaneous Drainage of the Pulmonary Abscess Under CT Guidance. (A) The procedure was performed in the CT suite, beginning with the administration of local anesthesia. (B) An axial CT image confirms the precise placement of the needle tip within the thick-walled abscess cavity, with clear avoidance of adjacent critical structures. (C) Aspiration of the cavity yielded thick, dark brown purulent fluid.

Antimicrobial therapy was guided by clinical assessment and was later refined by microbiological susceptibility results. During the intensive phase, the patient received intravenous meropenem (2 g every 8 h) in combination with doxycycline. After approximately two weeks, when the patient’s condition stabilized and vital signs improved, the regimen was transitioned to ceftazidime/avibactam for continued targeted therapy. Both meropenem and ceftazidime are first-line antibiotics recommended for melioidosis. After mechanical ventilation was discontinued, a follow-up CT scan revealed the formation of a pulmonary abscess, raising concern for a relapse of the infection. After a multidisciplinary team discussion, the decision was made to pursue source control. This was achieved through a successful CT-guided percutaneous drainage of the abscess. This minimally invasive technique (Fig. 3) was chosen for its high accuracy and capacity to reduce complications associated with drainage in anatomically challenging regions [9].

On admission, the patient remained tachypneic and febrile. To reduce oxygen consumption and mitigate patient–ventilator asynchrony, a neuromuscular blocking agent (rocuronium bromide) was administered in conjunction with deep sedation and analgesia. Our use of a 48-hour continuous infusion of NMBAs aligns with the 2024 American Thoracic Society clinical practice guideline for managing ARDS [10]. This intervention helped to alleviate dyspnea and facilitate protective ventilation.

The patient was diagnosed with septic cardiomyopathy, supported by elevated cardiac enzyme levels and reduced ejection fraction (see Supplementary Figure S8–9). Furthermore, he had already received substantial fluid resuscitation prior to transfer. On admission, signs of fluid overload were present, including moderate peripheral edema and a markedly elevated B-type natriuretic peptide (BNP) level. After achieving hemodynamic stability with vasoactive support, we implemented a strategy to maintain a continuous negative fluid balance. This approach aligns with the principles of conservative fluid management in critically ill patients [11] and is supported by the Extracorporeal Life Support Organization (ELSO) guidelines. The ELSO guidelines underscore the importance of this strategy, emphasizing that fluid overload is an independent predictor of mortality in patients receiving ECMO and that its late correction may not improve survival, which highlights the need for proactive fluid balance management [12].

A structured bundle-based approach (ABCDEF bundle) was applied as part of routine ICU care [13]. This included pain assessment and management, daily spontaneous awakening and breathing trials, protocolized sedation, delirium monitoring, early mobilization, and family engagement. Concurrently, the patient received standard sepsis treatments based on the SSC guidelines, including corticosteroids for shock, anticoagulation, enteral nutrition, along with other symptomatic therapies such as passive exercises and acupuncture [14].

### Follow-up and outcomes

The patient was liberated from VV-ECMO after 8 days and from invasive mechanical ventilation after 11 days. The percutaneous drainage of the pulmonary abscess marked a clear turning point in his recovery; afterward, his fever resolved, and inflammatory markers consistently declined. He made a substantial recovery of organ function over a hospitalization that lasted more than one month and was ultimately discharged without apparent neurologic or functional deficits. To prevent relapse, his treatment concluded with a multi-month course of oral antibiotics for eradication therapy using trimethoprim–sulfamethoxazole. He has remained clinically stable during regular outpatient follow-up.

### Ethics statement

The Ethics Committee of the Second Affiliated Hospital of Hainan Medical University reviewed and approved this case report. The patient’s legal representative provided written informed consent for the publication of this manuscript and the accompanying images.

## Discussion

The successful management of this case provides important insights for the clinical diagnosis and treatment of melioidosis complicated by severe ARDS. First, this case reaffirms the critical importance of maintaining a high index of suspicion for melioidosis in endemic regions. The patient's initial nonspecific presentation, mimicking common community-acquired pneumonia before rapidly progressing to fulminant sepsis, exemplifies why *B. pseudomallei* is notoriously known as the “great mimicker”. This underscores the need for clinicians in endemic regions such as Hainan to include melioidosis as a key differential diagnosis in patients presenting with severe community-acquired pneumonia or sepsis, especially when high-risk factors are present (e.g., diabetes, chronic alcoholism, or recent soil exposure during the rainy season) [4].

Second, our experience highlights the prudent interpretation required for advanced diagnostics like mNGS. The stark discrepancy between the high read count for T. whipplei (29,975 reads) and the low count for B. pseudomallei (22 reads) presented a significant diagnostic challenge. The key to resolving this was clinical correlation. T. whipplei is the agent of Whipple's disease, a chronic, indolent illness, which was entirely inconsistent with the patient's hyperacute, septic presentation [6]. In contrast, *B. pseudomallei* is a well-established cause of fulminant pneumonia and sepsis [2]. This case powerfully illustrates a critical limitation of mNGS: it detects nucleic acids, not necessarily viable, disease-causing organisms. The high *T. whipplei* signal may represent DNA translocation from the gut (a known commensal) due to increased intestinal permeability in septic shock, a phenomenon known as "leaky gut" [15]. Therefore, clinical reasoning, which weighs the pathogen’s known virulence against the patient's clinical syndrome, remains paramount and must override quantitative data alone to avoid diagnostic misdirection. In this patient, the clinical team correctly identified *B. pseudomallei*—which had a lower read count but high pathogenic potential—as the true causative agent based on the acute, fulminant disease course. This prevented diagnostic misdirection that could have resulted from overinterpreting the high read count of *T. whipplei*, thereby establishing the essential foundation for precise and effective treatment.

Third, this case contributes to the limited but growing body of evidence regarding the use of VV-ECMO in severe melioidosis-associated ARDS. Mortality in such cases remains exceedingly high [16]. While conventional lung-protective ventilation and prone positioning failed, the early initiation of VV-ECMO served as a crucial bridge to recovery. Its primary benefit extended beyond merely correcting refractory hypoxemia; it enabled an ultra-lung-protective strategy ("lung rest") with minimal driving pressure, mitigating ventilator-induced lung injury (VILI) and allowing time for targeted antimicrobials to take effect and the lungs to heal [17].

Furthermore, this case contributes to the limited but growing body of evidence regarding the use of VV-ECMO in severe melioidosis-associated ARDS. A recent scoping review by Jarrett et al. identified melioidosis as a key tropical infection suitable for VV-ECMO support, though the literature consists mainly of case reports and small series rather than large-scale studies [18]. Our experience aligns with earlier reports, such as the one by van der Geest et al., who described a successful outcome in a patient with a similar presentation of melioidosis-induced ARDS and refractory septic shock treated with VV-ECMO [19]. These cases underscore the life-saving potential of ECMO as a bridge to recovery when conventional support fails. However, more recent reports also highlight the complexity of these patients. For instance, Amali et al. described an ECMO-dependent patient with fulminant melioidosis who only recovered after receiving adjunctive interferon-gamma therapy to correct an underlying genetic immunodeficiency [20]. Compared to these reports, our case reinforces the concept that successful ECMO support is not merely a rescue therapy for hypoxemia but an integral component of a comprehensive, multimodal strategy. While our patient responded to a strategy focused on aggressive pathogen-directed therapy and definitive source control, the case by Amali et al. suggests that in select refractory cases, investigating and modulating the host's immune response may also be a critical, and perhaps necessary, component of care.

In addition, a structured antimicrobial strategy was fundamental to the successful outcome. Our approach was twofold. During the initial intensive phase, we administered meropenem, which is recommended by current treatment guidelines for severe melioidosis due to its strong in vitro activity against B. pseudomallei and favorable tissue penetration. Once clinical stabilization was achieved and microbiological confirmation was obtained, therapy was adjusted in accordance with susceptibility data and clinical response. Consistent with established treatment principles, the patient was subsequently transitioned to oral trimethoprim-sulfamethoxazole for prolonged eradication therapy to reduce the risk of relapse, which has been reported in up to 10%–20% of cases, closely associated with poor adherence to or premature discontinuation of eradication-phase therapy, as well as the presence of unaddressed latent infection foci. Evidence indicates that oral eradication therapy lasting less than 8 weeks significantly increases the risk of relapse, and adequate intravenous therapy during the intensive phase is also critical in preventing recurrence. This staged approach, combined with definitive source control via CT-guided drainage of the pulmonary abscess, was critical for both acute infection control and long-term cure.

Finally, certain limitations exist in the management of this case. First, as a single case report, the generalizability of these findings is inherently limited, and causal inferences regarding specific interventions cannot be established. Second, the complexity of the interventions (ECMO, multiple antibiotics, and abscess drainage) makes it impossible to isolate the individual contribution of each component to the patient's recovery. Third, while we concluded T. whipplei was a bystander, its potential role as a cofactor in an immunocompromised host cannot be definitively ruled out without further investigation.

## Conclusion

The successful management of this case demonstrates that melioidosis remains a critical potential cause of rapidly progressive severe pneumonia in tropical and subtropical regions. Rapid molecular pathogen detection using mNGS can provide important early clues before conventional test results are available, but interpretation of results must still be integrated with clinical features and epidemiological context. Long-term, staged, targeted antimicrobial therapy combined with VV-ECMO support and multidisciplinary collaboration provides valuable experience for improving patient outcomes.

## Author contributions

FYW, CZX and MRZ contributed equally to this work. They jointly conceived the study design and participated in the analysis of clinical data. YXP, YXX, and XZW provided important support in data collection and technical assistance. FYW drafted the manuscript in English. WWZ and YQX were involved in revising the manuscript critically for important intellectual content. All authors read and approved the final manuscript. All authors reviewed and approved the final version of the manuscript.

## CRediT authorship contribution statement

**Xiaozhi Wang:** Validation, Project administration. **Chengzhi Xie:** Writing – review & editing, Formal analysis, Data curation. **Mingrui Zhao:** Writing – review & editing, Resources, Conceptualization. **Yuxiang Pan:** Validation. **Yuxiang Xie:** Validation. **Fengyun Wang:** Writing – review & editing, Writing – original draft, Investigation, Formal analysis, Data curation, Conceptualization. **Weiwei Zhu:** Validation, Resources. **Yiqiang Xie:** Resources, Investigation.

## Consent for publication

The patient gave consent for publication, and consent for publication was written.

## Ethics approval and consent to participate

Not applicable (only standard care was performed).

## Funding

This work was supported by the 10.13039/501100004761Natural Science Foundation of Hainan Province (Grant No. 326MS0415, 2026), granted to Dr. Fengyun Wang.The project is supported by Hainan Province Clinical Medical Center and the National Key Clinical Specialty Program of China, awarded to the Department of Critical Care Medicine, The Second Affiliated Hospital of Hainan Medical University.

## Declaration of Competing Interest

The authors declare that they have no known competing financial interests or personal relationships that could have appeared to influence the work reported in this paper.

## Data Availability

No more data is available.
