# Supplementary material for: VV-ECMO–supported management of severe ARDS secondary to melioidosis sepsis: A case report and concise review
Source: IDCases. 2026 May 15;44:e02612. doi: 10.1016/j.idcr.2026.e02612 (PMC13217840; doi:10.1016/j.idcr.2026.e02612)
Supplement: Supplementary file 1 — Supplementary material [file mmc1.pdf]

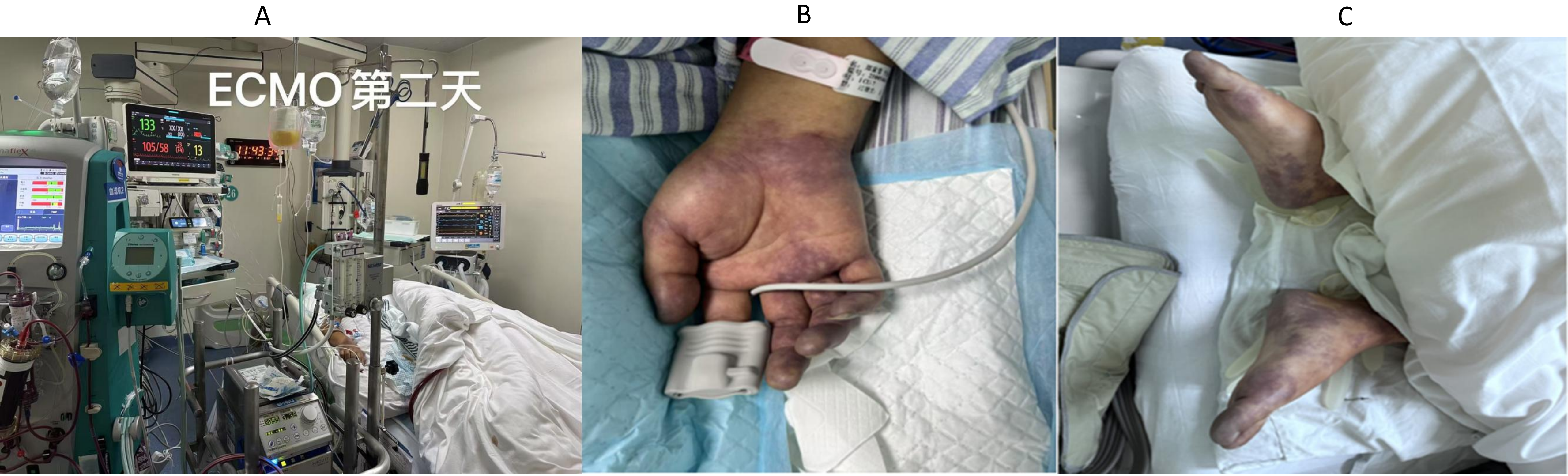

Figure S1. **Clinical condition of the patient on Day 2.**

(A) The patient in the intensive care unit receiving multi-organ support, including veno-venous extracorporeal membrane oxygenation (VV-ECMO), mechanical ventilation and continuous renal replacement therapy (CRRT). (B, C) Despite advanced life support, the patient's condition remained critical, with prominent signs of disseminated intravascular coagulation (DIC) manifesting as extensive ecchymosis on the dorsal aspects of his hands and feet.

Figure S2 ECMO parameters

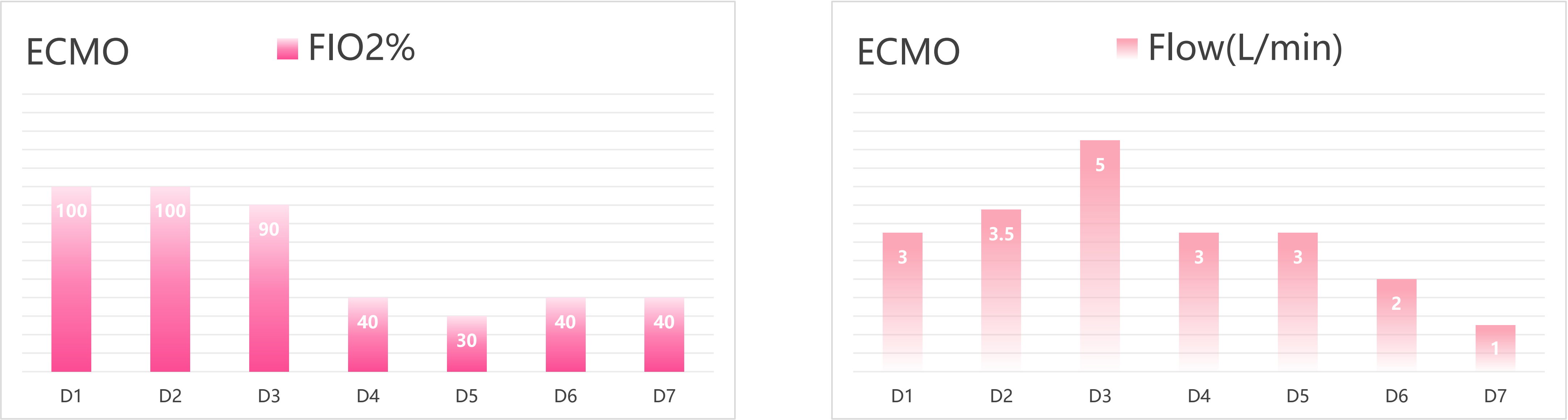

Figure S3 Inflammatory and infectious biomarkers

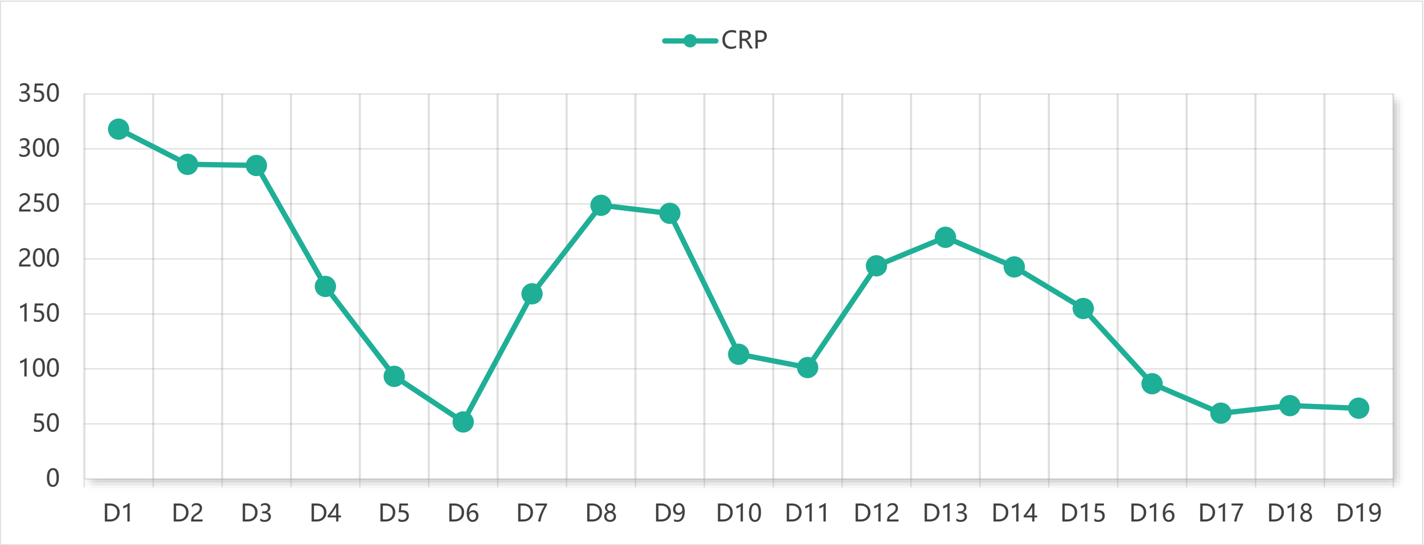

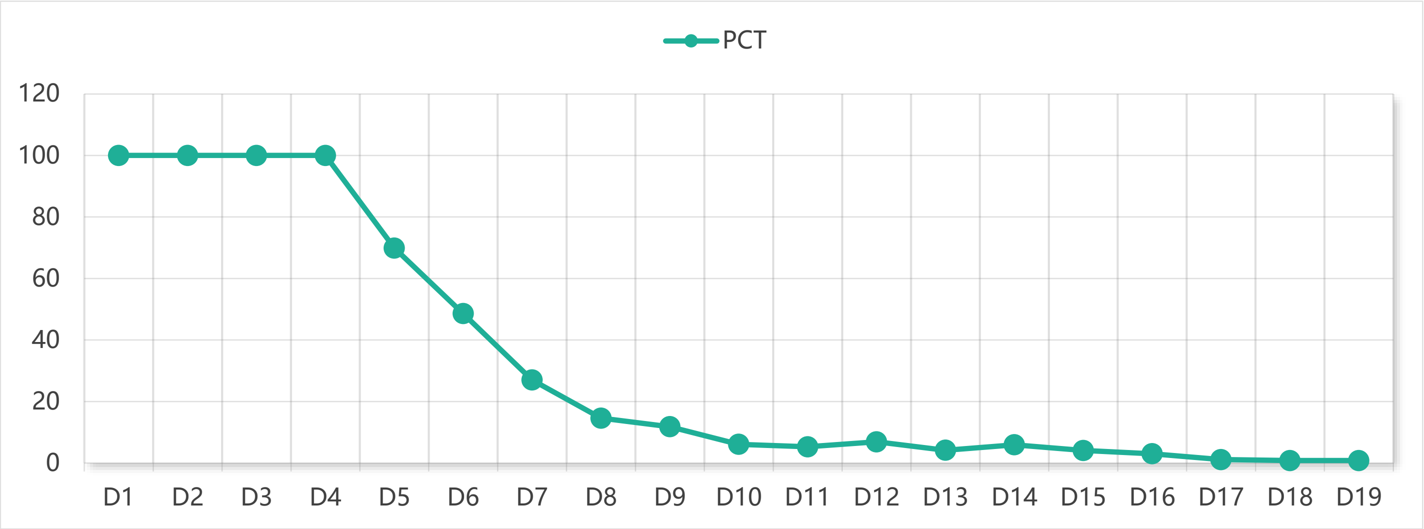

CRP: C-reactive protein, mg/L.
PCT: Procalcitonin, ng/ml.

Figure S4 Inflammatory and infectious biomarkers

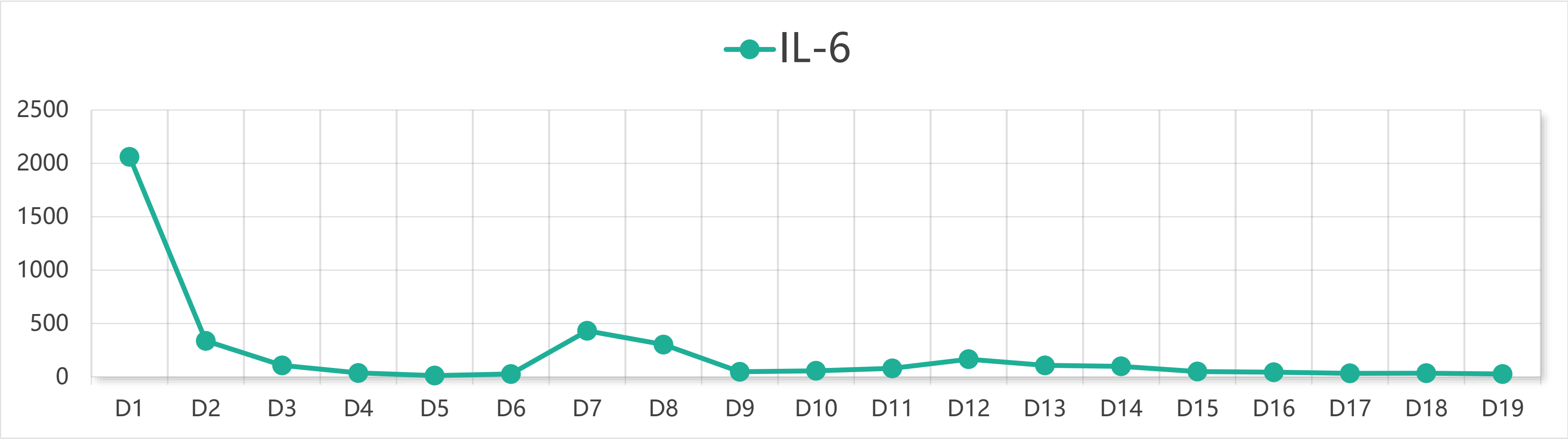

IL-6: Interleukin-6, pg/ml.

Figure S5 Oxygenation

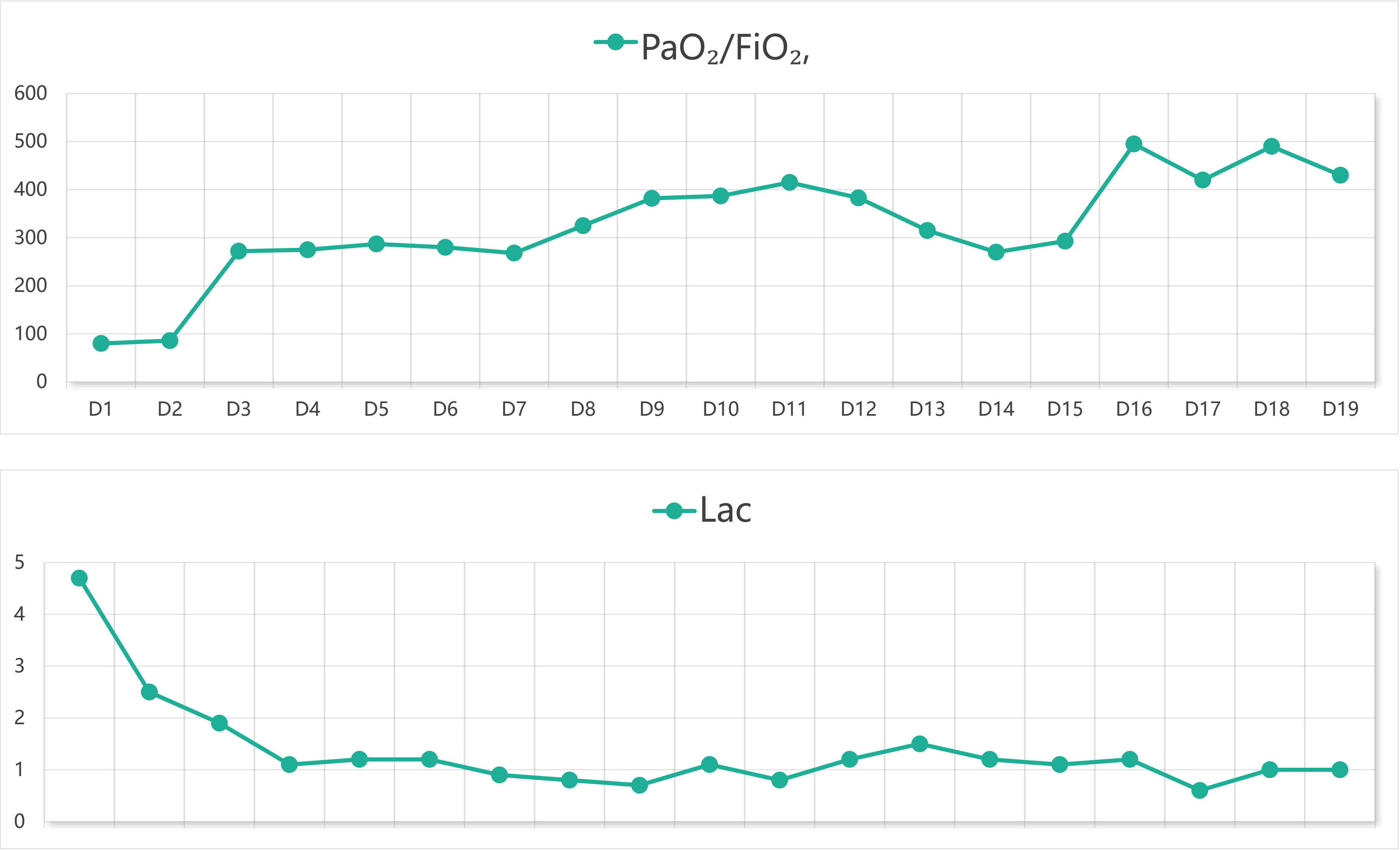

Lac: Lactate, mmol/L.

Figure S6 Kidney

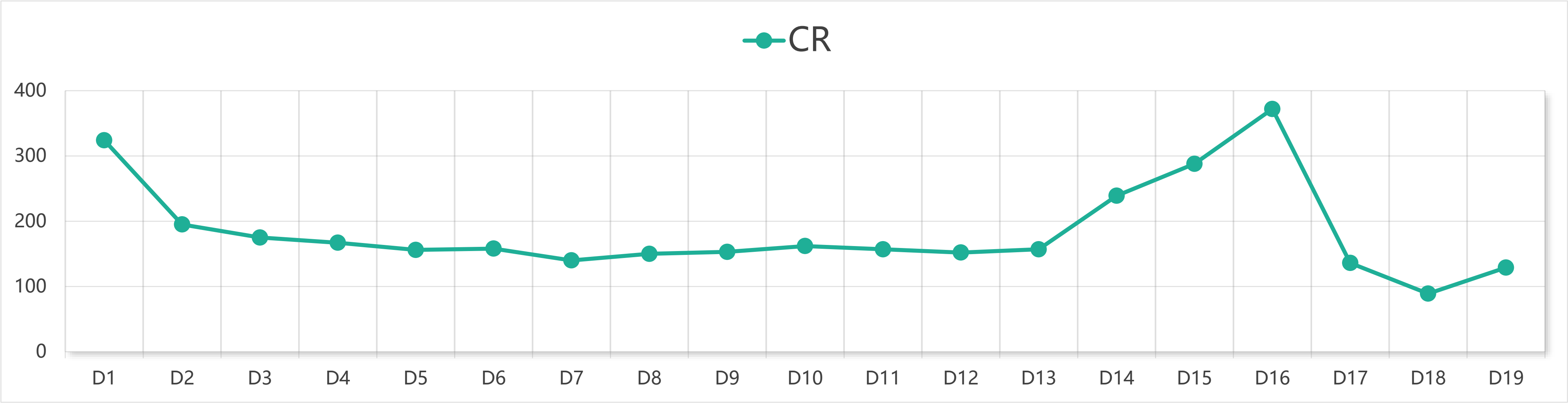

CR: Creatinine, μmol/L.

Figure S7 Liver

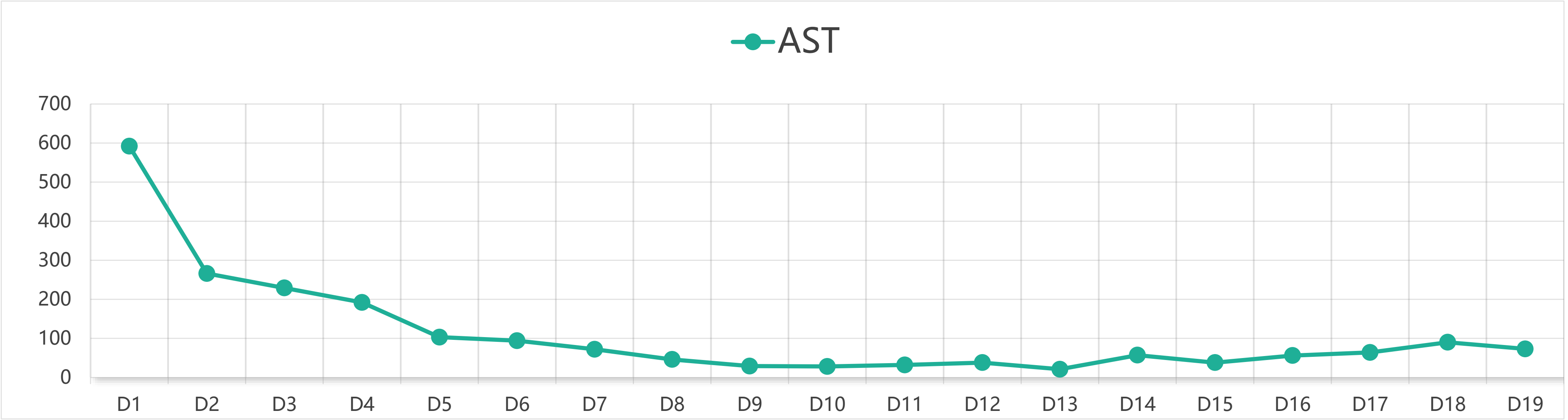

AST： Aspartate Aminotransferase, U/L.

Figure S8 Cardiac function

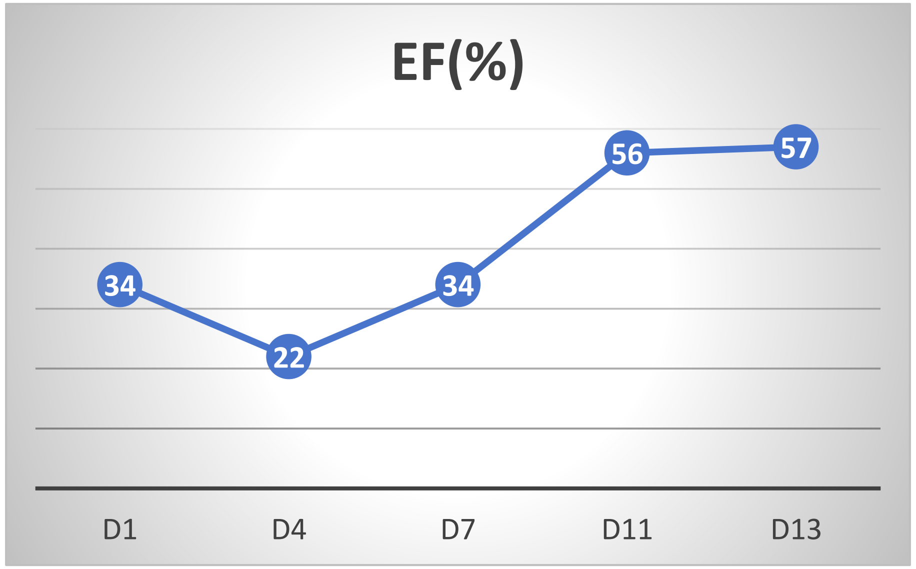

EF, Ejection Fraction, %

Figure S9 Heart

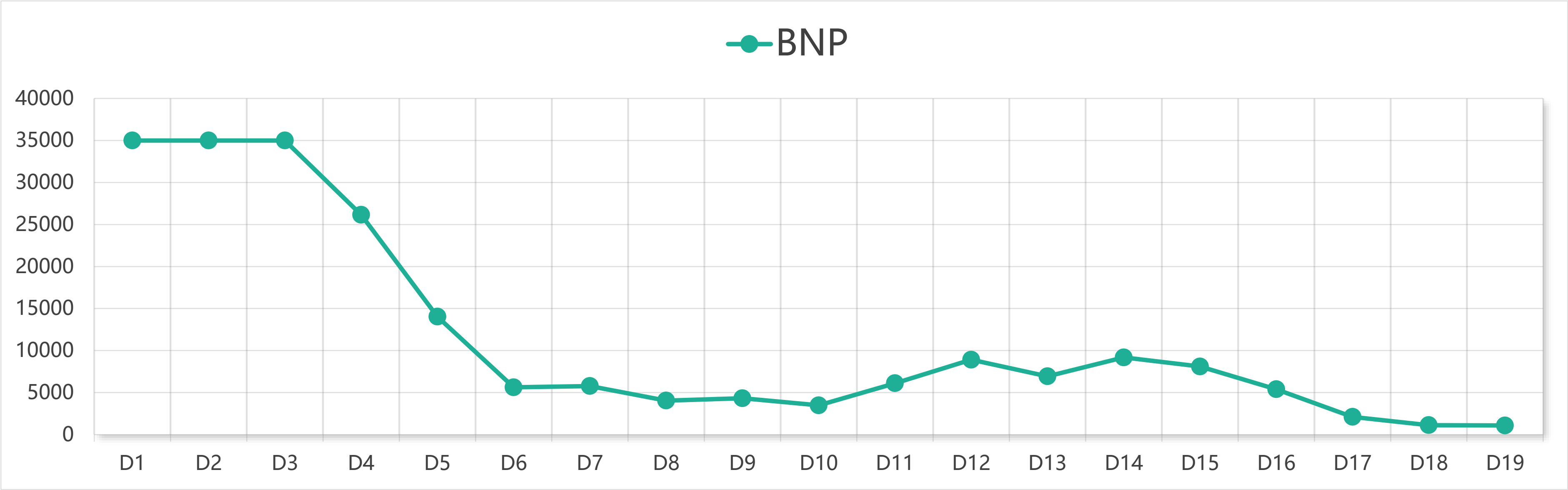

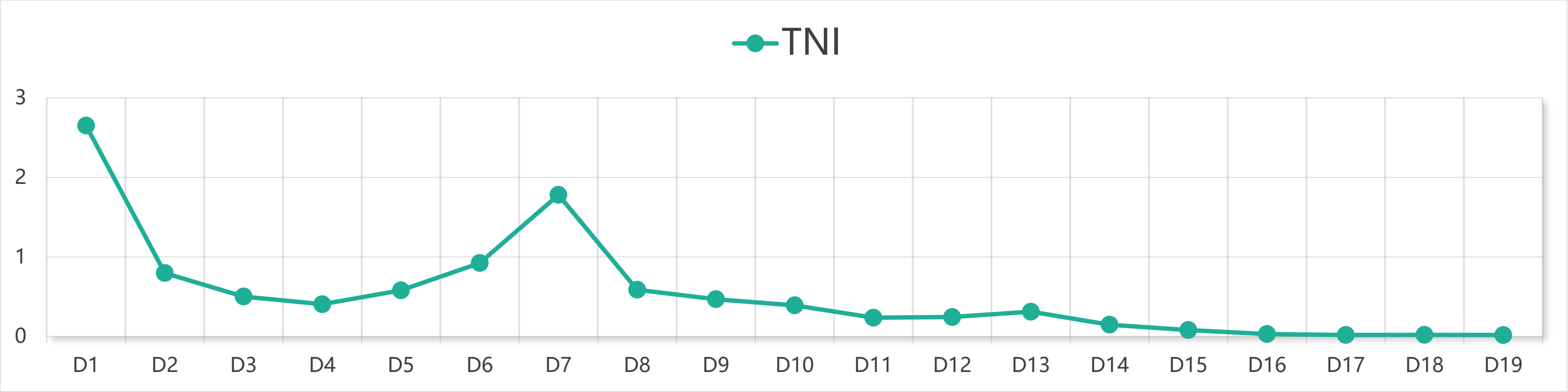

BNP: B-type Natriuretic Peptide, pg/ml.
TNI: Troponin I, ng/ml.

Figure S10 Coagulation

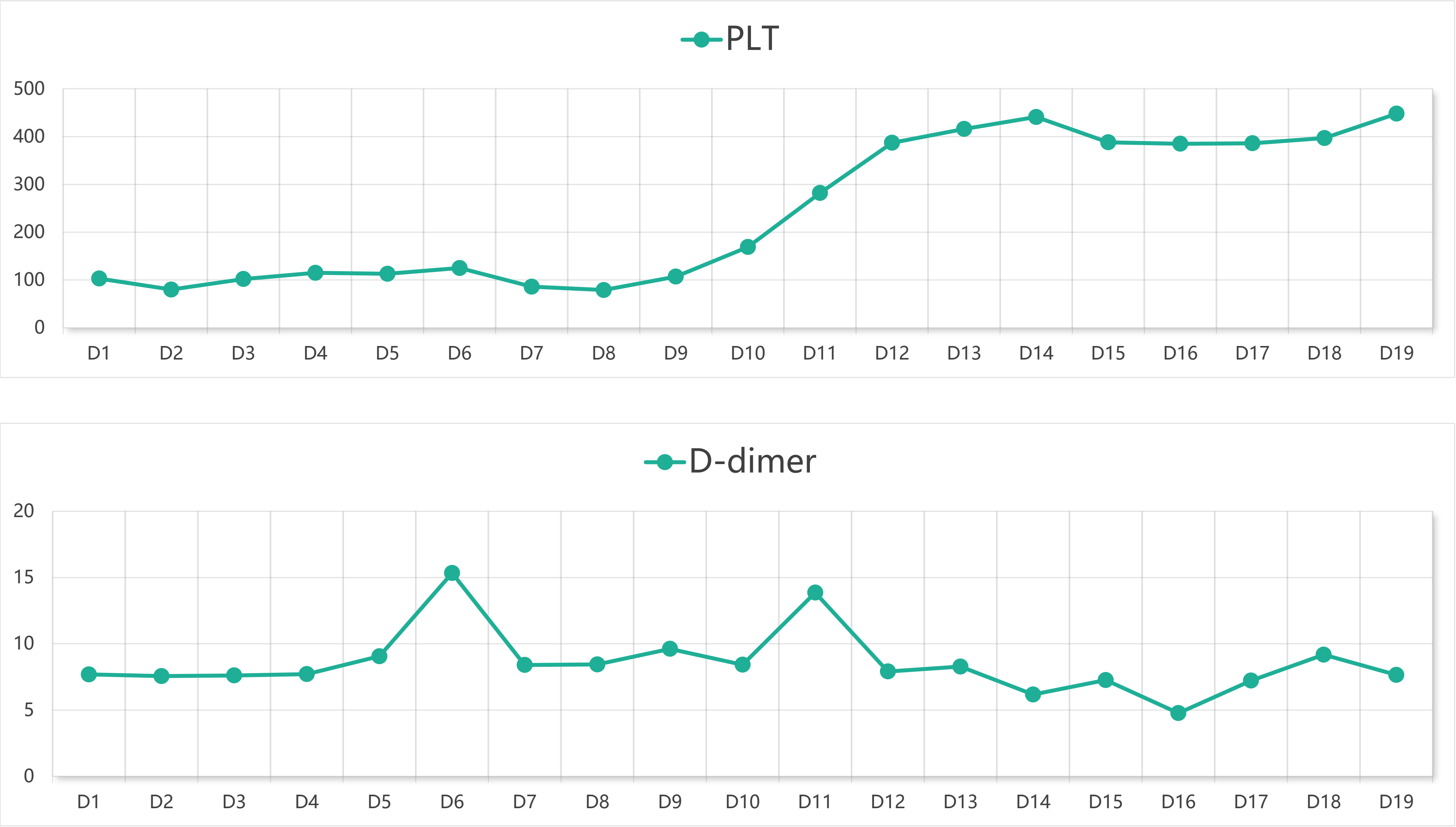

PLT： Platelet, 10^9/L.
D-II: D-dimer, μ/ml.

Figure S11 Coagulation

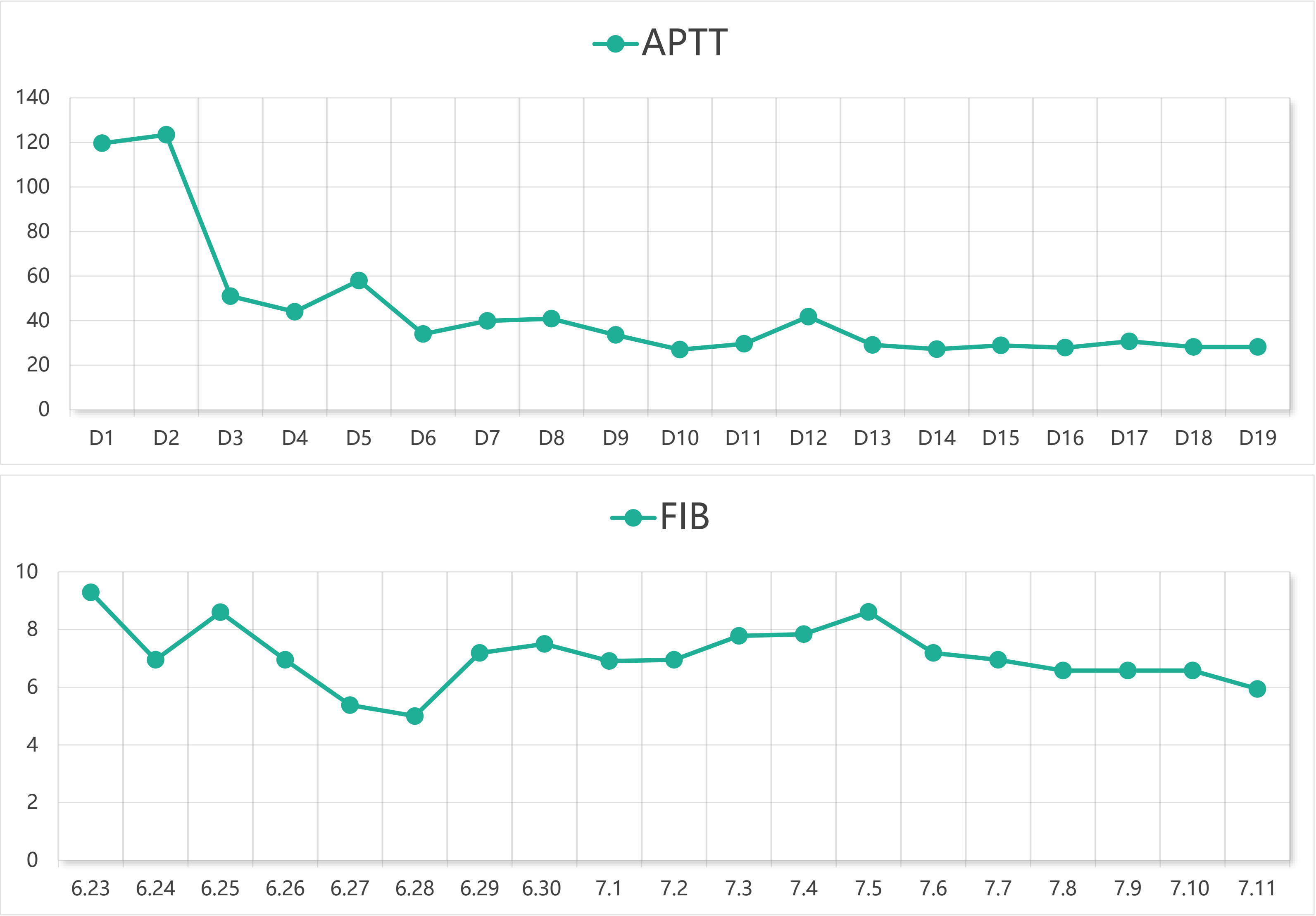

APTT： Activated Partial Thromboplastin Time, seconds.
Fibrinogen: FIB, g/L.
